# Emission Integral Effect on Non-Invasive Blood Glucose Measurements Made Using Mid-Infrared Passive Spectroscopic Imaging

**DOI:** 10.3390/s25061674

**Published:** 2025-03-08

**Authors:** Daichi Anabuki, Shiori Tahara, Hibiki Yano, Akira Nishiyama, Kenji Wada, Akiko Nishimura, Ichiro Ishimaru

**Affiliations:** 1 Graduate School of Science for Creative Emergence, Kagawa University, 2217-20 Hayashi-cho, Takamatsu, Kagawa 761-0396, Japan; 2 Faculty of Medicine, Kagawa University, 1750-1 Miki-cho, Kita, Kagawa 761-0793, Japan; 3 Faculty of Engineering and Design, Kagawa University, 2217-20 Hayashi-cho, Takamatsu, Kagawa 761-0396, Japan

**Keywords:** non-invasive blood glucose sensor, thermal emission spectroscopy, thermal radiation, Fourier-transform spectroscopy, mid-infrared spectroscopy

## Abstract

Living bodies emit mid-infrared light (wavelength band centered at approximately 10 µm) with a temperature-dependent intensity. Several studies have shown the possibility of measuring blood glucose levels using the mid-infrared emission of living bodies, and we have demonstrated non-invasive blood glucose measurements through distant wrist measurements (wavelength 8–14 µm) by mid-infrared passive spectroscopic imaging. However, it is not clear why blood glucose is detectable, as there is no formula that shows the effect of material thickness and concentration on emission intensity. In this study, we developed a principle for understanding glucose detection by proposing that an emission integral effect underpins the changes in emission intensity with substance thickness and absorption coefficient. We demonstrate the emission integral effect by measuring the spectral radiance of polypropylene with different thicknesses using mid-infrared passive spectroscopic imaging. The simulation results based on the emission integral effect indicate that in living bodies, dilute components such as glucose are easier to identify than components with high concentrations. Mid-infrared passive spectroscopic imaging offers potential innovative solutions for measuring various substances from a distance, with the emission integral effect acting as the basic working principle.

## 1. Introduction

Diabetes (fasting plasma glucose concentrations of ≥126 mg/dL) is a global health problem, with the number of cases increasing year by year [1,2]. The disease causes not only complications such as neuropathy and nephropathy, which reduce quality of life, but also atherosclerosis, which leads to death via heart disease and stroke [3]. However, the devices used to measure blood glucose levels in daily life are invasive, and therefore, both the treatment and prevention of diabetes involve pain. Although there is a need for non-invasive blood glucose sensors, none has yet been put into practice.

Many studies on non-invasive blood glucose measurements have used near-infrared spectroscopy, which has the advantage of high transmission through human skin [4,5,6,7,8]. However, near-infrared spectroscopy detects glucose as a vibrational overtone that has a weak absorption intensity. In addition, the prominent absorption peak of glucose at 1.69 µm overlaps with the broad absorption peak of water in the 1.45–1.79 µm range. Quantitative blood glucose measurements are, therefore, susceptible to changes in the skin moisture content. As a solution to this problem, previous research has implemented neural networks to improve analysis [9]. In contrast to near-infrared spectroscopy, mid-infrared spectroscopy can obtain spectral characteristics unique to various substances in the “fingerprint region” at approximately 10 µm [10,11]. Moreover, the intensity of the absorption peak is strong in the mid-infrared range because the fundamental vibrations of many materials occur within this band. However, mid-infrared light penetrates only as deep as the stratum corneum because of strong absorption by skin moisture [12]. In previous research, the measurement site has been limited to the oral mucosa [13]. Taking advantage of the spectral information that can be derived from mid-infrared light emitted by living bodies, we aim to realize non-invasive blood glucose measurements made using mid-infrared passive spectroscopic imaging [14].

Mid-infrared light is emitted from the skin surface with an intensity that depends on body temperature, as described by Planck’s law. Thermographs and non-contact thermometers detect the integrated intensity of this emission at a specific wavelength band and convert it into temperature. Non-invasive blood glucose sensors based on emission measurements have been proposed, although many studies did not use spectroscopy, instead using bandpass filters to detect only the emissions at the wavelengths needed to identify glucose [15,16,17,18]. Because of their low sensitivity, these sensors were, in many cases, limited to measurements of the tympanic membrane, which is minimally affected by ambient light. In contrast, mid-infrared passive spectroscopic imaging provides spectral characteristics over a wide wavelength range of 8 to 13 µm. We previously demonstrated the potential of using mid-infrared passive spectroscopic imaging to make distant wrist measurements for non-invasive blood glucose measurements [14]. In this study, multiple measurements were taken in five healthy subjects before/after sugar intake. We found a high correlation between the emitted light intensity and the blood glucose levels obtained with a commercially available blood glucose sensor. However, no one has explained the principle by which blood glucose levels can be evaluated from emission measurements. Furthermore, in the field of spectroscopy, there are no studies that clarify how the emission intensity changes with substance thickness. To further advance the potential of mid-infrared passive spectroscopic imaging, we aimed to provide an explanation of how blood glucose levels can be detected from mid-infrared emission measurements and how the measured emission intensity changes with the substance thickness. In this study, we developed a principle for understanding glucose detection by proposing that an emission integral effect underpins the change in emission intensity with substance thickness and absorption coefficient.

## 2. Materials and Methods

### 2.1. Derivation of the Emission Integral Effect

**Figure 1:**
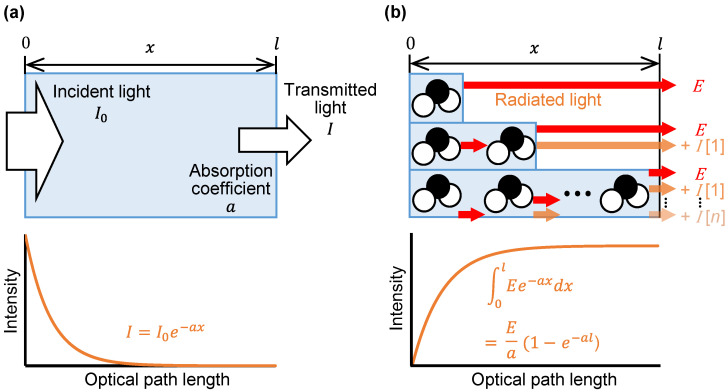
Schematic comparison of the Beer–Lambert law and emission integral effect: (**a**) Beer–Lambert law in infrared spectroscopy; (**b**) model of the emission integral effect in mid-infrared passive spectroscopic imaging. The blue bars indicate the thickness of the sample with only one molecule present, two molecules present, and n molecules present. As the thickness increases, the emission intensity of a single molecule (*E*) decays to *I* [*n*] depending on the number n of molecules present.

Figure 1 compares the Beer–Lambert law in infrared spectroscopy with the emission integral effect in mid-infrared passive spectroscopic imaging. In infrared spectroscopy, the transmitted light intensity decreases exponentially as the substance thickness increases. The Beer–Lambert law quantifies this phenomenon by assigning a unique absorption coefficient to each wavelength, as shown in Figure 1a. In the case of measuring emission, such as by mid-infrared passive spectroscopic imaging, the intensity of the detected light increases with increasing substance thickness, and we propose that the emission integral effect underpins this phenomenon. Molecules that make up a substance emit light through molecular vibration. If we consider these molecules as the light source, then the intensity of light emitted by one molecule is reduced according to the Beer–Lambert law because of absorption by other molecules. Because there are many molecules in the substance, we observe the integrated intensity of the attenuated emission light, as shown in Figure 1b. We express this emission integral effect as an integration of the Beer–Lambert law:

(1) $$\sum_{i=0}^{n}E_{i}=\int_{0}^{l}Ee^{-ax}dx=\frac{E}{a}(1-e^{-al})$$

Here, *E* is the intensity of light emitted by an individual target molecule, a is the absorption coefficient, and *l* is the thickness of the substance. As shown in Figure 1b, the intensity of emitted light in mid-infrared passive spectroscopic imaging, therefore, increases with increasing substance thickness following a logarithmic function.

### 2.2. Experiments Using Polypropylene to Demonstrate the Emission Integral Effect

This emission integral effect assumes that the emission intensity of each molecule and the number of molecules present in the depth direction are constant. In the demonstration experiments based on this assumption described in the Results Section, we used polypropylene because its thickness is easy to control. The emitted intensity E is considered to be a temperature variable based on Planck’s law. Because the sample was measured at room temperature, with it in thermal equilibrium, the emitted intensity E of all the molecules is constant. Because each measurement takes approximately 10 s, the room temperature fluctuations over the several minutes required for the experiment will not have a large effect. We measured the temperature of the sample with a thermometer (resolution: 0.1 °C; CT-05SD, CUSTOM Corp., Tokyo, Japan) before the measurements. For macroscopic phenomena, such as with the polypropylene used in this demonstration experiment, a small difference in the number of molecules is not expected to have any effect.

### 2.3. Internal Optics of the Apparatus Capable of Mid-Infrared Passive Spectroscopic Imaging

**Figure 2:**
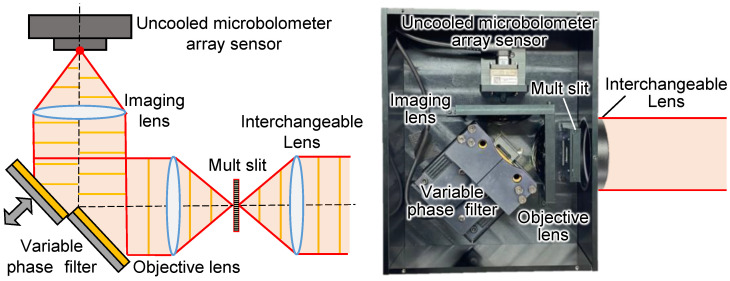
Internal optics of an imaging-type two-dimensional Fourier spectrometer.

The spectral radiance of the sample was obtained using an imaging-type two-dimensional Fourier spectrometer that we developed, as shown in Figure 2 [19,20]. The proposed apparatus consists of two lenses (germanium, lens diameter 50 mm, focal length 25 mm), an uncooled microbolometer array sensor (array size: 320 × 256 pixels, pixel pitch: 12 µm, sensitivity range: 7.5 to 13.5 µm, Boson 320, FLIR Systems Inc., Wilsonville, OR, USA), and a phase-variable filter. The phase-variable filter is composed of a fixed mirror and a movable mirror, which gives a phase difference to half of the object’s light beam. This makes it possible to obtain two-dimensional spectral characteristics from successive images. The measurement range of this apparatus can be changed using interchangeable lenses, and the same lens as in the apparatus (germanium, lens diameter 50 mm, focal length 25 mm) was used in this experiment. In addition, a multi-slit was placed on the conjugate plane of the detector to improve interference-pattern visibility [21]. This made the apparatus capable of mid-infrared passive spectroscopic imaging, even with weak radiation emitted from samples at room temperature.

### 2.4. Measurement of Spectral Radiance and Radiance

We measured the spectral radiance using an imaging-type two-dimensional Fourier spectrometer and the radiance using a mid-infrared camera. In the two-dimensional data, we averaged the intensity of 30 × 30 pixels in the polypropylene. Pixels near the center were selected to reduce the effect of optical aberration.

### 2.5. Simulation of Spectral Radiance at Different Substance Thicknesses

Because it is difficult to determine *E* in Equation (1), we simulated the intensity changes with thickness changes in steps of 0.04 mm using the experimentally obtained emission intensity of 0.04 mm thick polypropylene. The emission intensity of the 0.04 mm thick polypropylene was used to simulate the intensity change as follows:

(2) $$E_{0.04}+E_{0.04}e^{-0.04a}+E_{0.04}e^{-0.08a}\cdots +E_{0.04}e^{-la}$$

Here, $E_{0.04}$ represents the luminescence intensity of 0.04 mm thick polypropylene and *l* is the thickness (a multiple of 0.04) that we want to estimate. Linear interpolation was used to simulate the intensities, which were compared with the experimental results.

### 2.6. Simulation of Spectral Radiance at Dermal Thickness

In the simulations, mid-infrared passive spectroscopic imaging was conducted on the wrist of a healthy person following a previously described experimental approach [14]. The simulations were performed using Equation (1), with the absorption coefficient a set to the value obtained from the absorption spectrum of glucose acquired by Fourier-transform infrared spectroscopy. Because *E* is unknown, and the goal is to see whether the spectral radiance attains a saturation-like status at a dermal thickness, the spectral radiance was set to converge to 1.

## 3. Results

### 3.1. Verification of the Emission Integral Effect on Radiance

**Figure 3:**
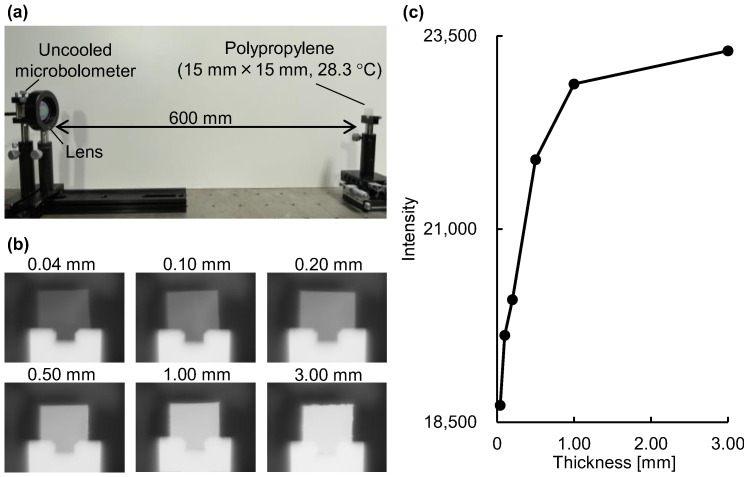
Verification of the emission integral effect on the radiance of polypropylene: (**a**) experimental setup; (**b**) mid-infrared images of polypropylene with different thicknesses. The intensity range is the same for all images; (**c**) dependence of radiance (average intensity of 100 images) on substance thickness.

To verify the emission integral effect, we measured the radiance of polypropylene at room temperature using an uncooled microbolometer attached to an imaging-type two-dimensional Fourier spectrometer at a distance of 600 mm (Figure 3a). A lens (germanium, lens diameter 25 mm, focal length 25 mm) was placed in front of the uncooled microbolometer. The uncooled microbolometer detects the intensity of emitted light at a given wavelength band as radiance, similar to the modus operandi of thermographs and non-contact thermometers. As shown in Figure 3b, the radiance in the polypropylene region of the acquired mid-infrared images increased as the polypropylene thickness increased. In agreement with Equation (1), the quantification of the radiance demonstrated that the intensity of emitted light increased following a logarithmic-like function with increasing substance thickness (see Figure 1b and Figure 3c).

### 3.2. Verification of the Emission Integral Effect on Spectral Radiance

**Figure 4:**
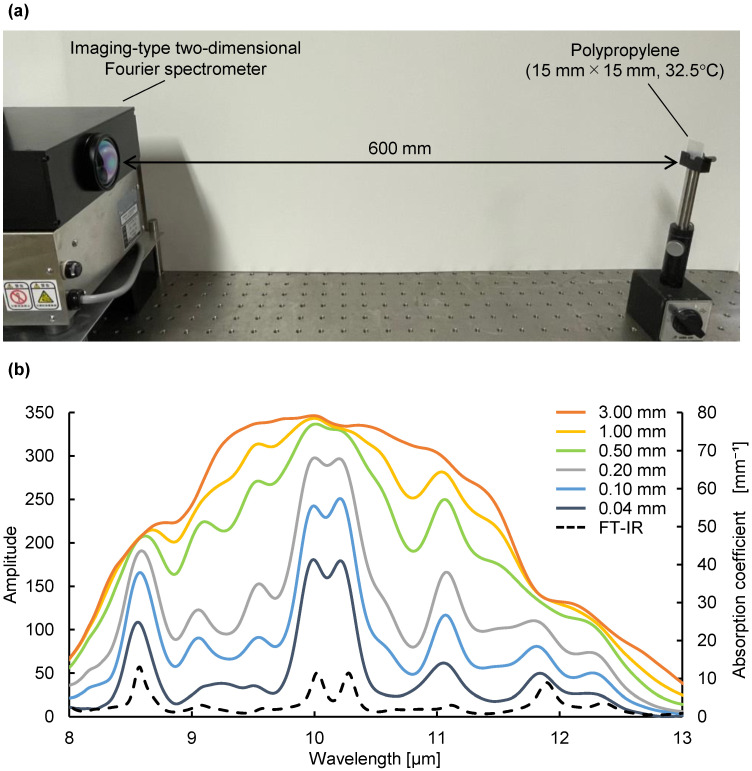
Verification of the emission integral effect on spectral characteristics of polypropylene using mid-infrared passive spectroscopic imaging: (**a**) experimental setup; (**b**) amplitude (left axis) and absorption coefficient (right axis) of polypropylene. The solid lines show the spectra of polypropylene of different thicknesses obtained using mid-infrared passive spectroscopic imaging. The black dashed line is a plot of the absorption coefficient of polypropylene obtained using Fourier-transform infrared spectroscopy.

**Figure 5:**
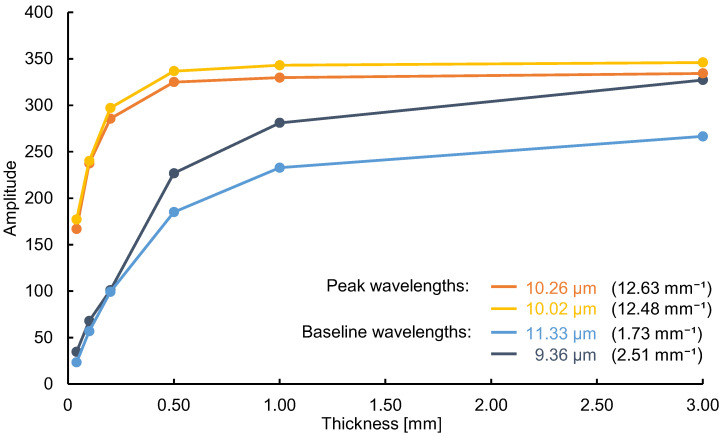
Spectral radiance of polypropylene at various substance thicknesses. The spectral radiance at the peak wavelengths (10.02 and 10.26 µm) changes at a higher rate than that at the baseline wavelengths (9.36 and 11.33 µm). Data correspond to those shown in Figure 3b. The absorption coefficients (mm^−1^) are shown in parentheses.

Next, we verified that not only the thickness of the substance, but also the absorption coefficient at each wavelength, is a variable in the emission integral effect. Because a large absorption coefficient means a large emission intensity, a large change in emission intensity can be expected at peak wavelengths with large absorption coefficients. Using mid-infrared passive spectroscopic imaging, we obtained the spectral radiance of polypropylene, which represents the intensity of emitted light at each wavelength. As shown in Figure 4a, the same polypropylene substances used in the experiment to prepare Figure 3 were measured from a distance of 600 mm. Figure 4b shows that the emission intensity at each wavelength increased as the substance thickness increased, and Figure 5 shows that the rate of this increase was greater at the peak wavelengths of 10.02 and 10.26 µm (where a is large) than at the baseline wavelengths of 9.36 and 11.33 µm (where a is small). Moreover, Table 1 shows the ratio of the mean values (values in Figure 4) to three times the standard deviation for the 30 replicates of this experiment. Approximately 99.7% of the data are expected to vary within ±6% of the mean, making this a precise and repeatable measurement. Therefore, consistent with Equation (1) and Figure 1b, the emission integral effect depends not only on the thickness of the substance, but also on the absorption coefficient at each wavelength. The spectral characteristics of the 3.00 mm thick polypropylene clearly show that a saturation-like behavior occurred, in which the intensities at the peak and baseline wavelengths were no longer significantly different when the intensity increased due to the emission integral effect became too great (Figure 4b). Therefore, when using mid-infrared passive spectroscopic imaging, it should be easier to identify the components of thinner materials and/or those with small absorption coefficients.

## 4. Discussion

### 4.1. Verification of the Emission Integral Effect Through Simulation

**Figure 6:**
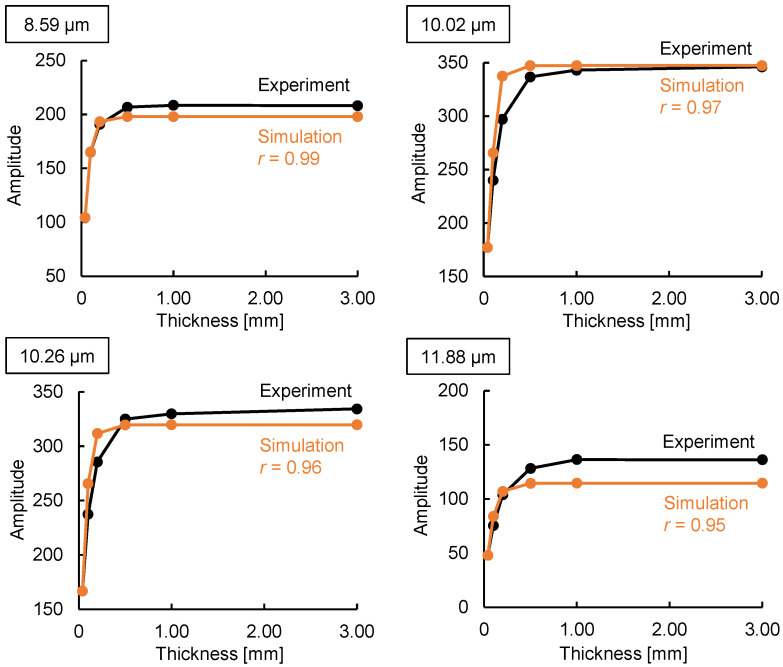
Simulation of the emission intensity of polypropylene at various substance thicknesses. The simulations were performed at peak wavelengths of 8.59, 10.02, 10.26, and 11.88 µm. The experimental data correspond to those shown in Figure 3b.

To quantitatively verify the emission integral effect, we compared the simulated and experimental results obtained for the intensity of light emitted by polypropylene with different thicknesses. Macroscopically, incident light on an object is split into three paths: reflection, absorption, and transmission. This means that incident light intensity = absorption intensity + reflection intensity + transmission intensity. When measuring the absorption coefficient (absorption) using common infrared spectroscopy techniques such as Fourier-transform infrared spectroscopy, Fresnel reflection occurs on the surface of the sample. Because transmission intensity = incident light intensity − absorption intensity − reflection intensity, the apparent absorption coefficient increases because of reflection as well as absorption. However, the emission integral effect models the phenomenon within the sample, so the emission intensity (incident light intensity) is not affected by Fresnel reflection. Therefore, the simulations were performed at peak wavelengths of 8.59, 10.02, 10.26, and 11.88 µm, where the effect of the Fresnel reflection is small because of the strong emission intensity (absorption intensity). The correlations between the simulation data and experimental data were high, 0.99 at 8.59 µm, 0.97 at 10.02 µm, 0.96 at 10.26 µm, and 0.95 at 11.88 µm (Figure 6), which verified the model.

### 4.2. Emission Integral Effect on Blood Glucose at the Wrist

**Figure 7:**
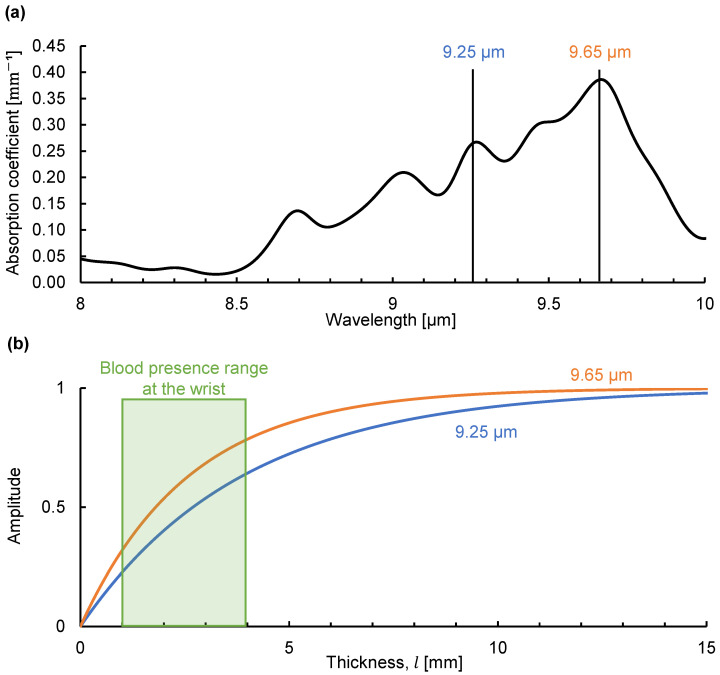
Simulation of the emission integral effect on blood glucose at the wrist: (**a**) absorption coefficient of glucose in a 100 mg/dL glucose solution obtained using Fourier-transform infrared spectroscopy; (**b**) simulation of the change in spectral radiance at the peak wavelengths (9.25 and 9.65 µm) at various solution thicknesses (*l*).

Finally, we simulated the emission integral effect on the light emitted by blood glucose at the wrist. The fasting blood glucose concentration in healthy people is less than 100 mg/dL [22]. Figure 7a shows the absorption coefficient of glucose in a 100 mg/dL aqueous glucose solution determined using Fourier-transform infrared spectroscopy. Using this absorption coefficient, we simulated the change in the emission intensity of glucose at the peak absorption wavelengths of 9.25 and 9.65 µm as the solution thickness increased, as shown in Figure 7b. Because blood circulates at a fast rate, we assume that the emission intensity E (which depends on temperature) of the glucose molecules in each blood vessel is constant. We consider the thickness of the dermis at the wrist (which is approximately 1–2 mm) where many capillaries are present as a rough estimate of the capillary thickness at the wrist [23,24]. Large vessels such as the radial artery are in the range of 1 to 2 mm in diameter [25]. We consider the thickness of the tissue containing blood to be 1 to 4 mm at the wrist. Within this range, the simulated emission intensity at each peak wavelength was in an increasing process and unaffected by saturation-like behavior, indicating that glucose identification is feasible. This should not be affected even if the intensity increases by several times with variations in the skin thickness. Therefore, the emission integral effect favors the detection of dilute analytes such as blood glucose. We believe that this phenomenon is one of the reasons why non-invasive sensors measuring emitted radiation can detect blood glucose. Using mid-infrared passive spectroscopic imaging, we aim to extend blood glucose detection to include the two-dimensional monitoring of multiple people from a distance. One of the challenges in achieving this goal is the variation in skin thickness arising from differences between individuals and measurement sites [26]. We have reported that there are individual differences in the results of wrist measurements using mid-infrared passive spectroscopic imaging [14]. We believe that these differences can be compensated for by focusing on the intensity ratio of glucose emission peaks and accounting for the emission integral effect.

## 5. Conclusions

We investigated the principle of non-invasive blood glucose detection by examining the relationship between the substance thickness and the intensity of emitted mid-infrared radiation. We derived and quantitatively evaluated the emission integral effect describing how the substance thickness and absorption coefficient affect the intensity of emitted light. We then demonstrated the validity of the emission integral effect by comparing the experimental and simulated emission intensities of polypropylene using mid-infrared passive spectroscopic imaging. Our simulations also demonstrate that the emission integral effect is advantageous for the identification of dilute components such as glucose by mid-infrared passive spectroscopic imaging. Therefore, the emission integral effect may be a factor enabling non-invasive blood glucose measurements from emission measurements. Additionally, the emission integral effect may be useful for the correction of individual differences in wrist measurements using mid-infrared passive spectroscopic imaging. Future research should investigate the influence of changes such as temperature gradients and non-uniformity of the tissue structure on the results. Furthermore, the emission integral effect should be verified in a multi-layer model to establish whether the single-layer model used in this study can be applied to human tissue. Mid-infrared passive spectroscopic imaging has the potential for innovative solutions for measuring various substances from a distance, and we expect the emission integral effect to be the basic working principle.

## Figures and Tables

**Table 1:** Mean (of the values shown in Figure 4) and 3σ ratio for 30 replicates.

Ratio of 3σ to the Mean (%)						
	0.04 mm	0.10 mm	0.20 mm	0.50 mm	1.00 mm	3.00 mm
9.36 µm	4.22	4.57	5.58	4.33	3.65	2.81
10.02 µm	3.79	4.50	3.08	3.72	2.33	2.27
10.26 µm	5.77	4.58	3.93	3.39	2.57	3.13
11.33 µm	5.57	5.41	5.32	3.14	3.35	3.63

## Data Availability

The data presented in this study are available upon request from the corresponding author.
